# A Full-Length Infectious cDNA Clone of Zika Virus from the 2015 Epidemic in Brazil as a Genetic Platform for Studies of Virus-Host Interactions and Vaccine Development

**DOI:** 10.1128/mBio.01114-16

**Published:** 2016-08-23

**Authors:** Konstantin A. Tsetsarkin, Heather Kenney, Rubing Chen, Guangping Liu, Hasmik Manukyan, Stephen S. Whitehead, Majid Laassri, Konstantin Chumakov, Alexander G. Pletnev

**Affiliations:** a Laboratory of Infectious Diseases, National Institute of Allergy and Infectious Diseases, National Institutes of Health, Bethesda, Maryland, USA; b Department of Pathology, Institute for Human Infections and Immunity, University of Texas Medical Branch, Galveston, Texas, USA; c Center for Biologics Evaluation and Research, U.S. Food and Drug Administration, Silver Spring, Maryland, USA

## Abstract

An arthropod-borne virus, Zika virus (ZIKV), has recently emerged as a major human pathogen. Associated with complications during perinatal development and Guillain-Barré syndrome in adults, ZIKV raises new challenges for understanding the molecular determinants of flavivirus pathogenesis. This underscores the necessity for the development of a reverse genetic system based on an epidemic ZIKV strain. Here, we describe the generation and characterization in cell cultures of an infectious cDNA clone of ZIKV isolated from the 2015 epidemic in Brazil. The cDNA-derived ZIKV replicated efficiently in a variety of cell lines, including those of both neuronal and placental origin. We observed that the growth of cDNA-derived virus was attenuated compared to the growth of the parental isolate in most cell lines, which correlates with substantial differences in sequence heterogeneity between these viruses that were determined by deep-sequencing analysis. Our findings support the role of genetic diversity in maintaining the replicative fitness of viral populations under changing conditions. Moreover, these results indicate that caution should be exercised when interpreting the results of reverse-genetics experiments in attempts to accurately predict the biology of natural viruses. Finally, a Vero cell-adapted cDNA clone of ZIKV was generated that can be used as a convenient platform for studies aimed at the development of ZIKV vaccines and therapeutics.

## IMPORTANCE

The availability of genetic tools and laboratory models determines the progress in understanding mechanisms of virus emergence and pathogenesis. Recent large-scale outbreaks of Zika virus (ZIKV) that were linked to complications during perinatal development and Guillain-Barré syndrome in adults emphasize the urgency for the development of a reverse-genetics system based on an epidemic ZIKV strain. Here, we report a stable infectious cDNA clone for ZIKV isolated during the 2015 epidemic in Brazil, as well as a Vero cell-adapted version of it, which will be used for virus-host interaction studies and vaccine development.

## INTRODUCTION

Recently an arthropod-borne virus, Zika virus (ZIKV), previously known only to a small number of infectious disease specialists, has been brought from obscurity into the spotlight of public and scientific attention (1). Besides the explosive nature of the ZIKV outbreaks that have caused millions of infections in new geographic ranges (2, 3), recent interest has been sparked by the mounting body of evidence linking ZIKV infection in pregnant women with severe defects in fetus development, abortions, and stillbirths (4–6). Moreover, the increased incidence of nervous system abnormalities in adult patients living in ZIKV-affected areas has linked this virus to severe diseases, including Guillain-Barré syndrome. Finally, unlike other well-studied members of the mosquito-borne group within the *Flavivirus* genus, ZIKV was able to establish long-term persistent infection in vertebrate hosts and utilize a sexual mode of transmission (7–9), posing new challenges for controlling outbreaks caused by this virus.

**FIG 1:**
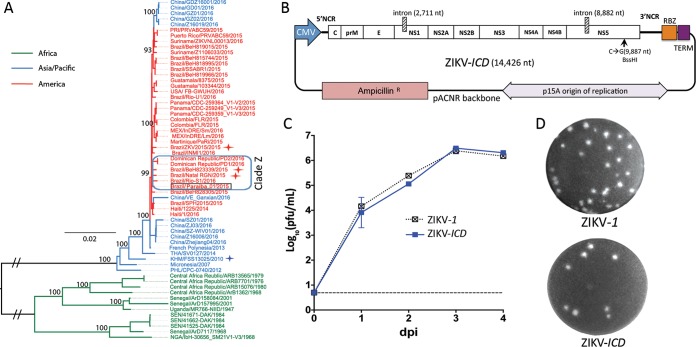
Reverse-genetics system for epidemic strain of ZIKV. (A) Maximum-likelihood phylogenetic tree with bootstrap values for the Paraiba_01/2015 strain (highlighted with a black box) and 60 representative ZIKV isolates. Color coding emphasizes the geographic origins of the strains. ZIKV strains associated with human microcephaly cases (Natal RGN, ZKV2015, and BeH823339) are highlighted with red stars. Strain FSS13025 is highlighted with a blue star. (B) Schematic map of ZIKV-*ICD* plasmid DNA. NCR, noncoding region; RBZ, antigenomic ribozyme of HDV; TERM, poly(A) signal/RNA-*polII* terminator. (C) Growth kinetics of ZIKV-*ICD* and ZIKV-*1* after plasmid DNA transfection into Vero cells. The dashed line indicates the limit of virus detection (0.7 log_10_ PFU/ml). (D) Plaque morphology of ZIKV-*ICD* and ZIKV-*1* in Vero cells at 4 days postinfection (dpi).

The availability of appropriate genetic tools and laboratory models typically determines the progress in understanding the mechanisms of virus emergence and severity of disease caused by virus infection. Such tools are also instrumental for studies aimed at the development of effective vaccines and therapeutics. In the case of ZIKV, substantial progress was made recently in establishing tissue culture, mosquito, and small animal models to study ZIKV pathogenesis (10–13). In addition, an infectious cDNA (ICD) clone for strain FSS13025, isolated in 2010 in Cambodia, has recently been described (14). This strain belongs to the Asian ZIKV lineage and is related to viruses that caused large-scale outbreaks in the South Pacific in 2013–2014 and the ongoing epidemic in South and Central Americas (Fig. 1A). However, there is a substantial sequence divergence between strain FSS13025 and the American sublineage, limiting the relevance of this clone for studies of ZIKV neurovirulence and pathogenesis. For instance, strain FSS13025 differs by 19 amino acid changes from the Brazilian ZIKV strains that are associated with the development of microcephaly in the human fetus (14). To overcome these constraints, we developed and characterized a full-length ICD clone of a ZIKV isolate obtained in 2015 from a patient in Brazil.

## RESULTS

### Paraiba_01/2015 strain.

The Paraiba_01/2015 strain of ZIKV was isolated in the state of Paraiba (Brazil) in 2015 from serum of a febrile female. The full-length genome sequence of Paraiba_01/2015 was generated using Sanger sequencing technology. Phylogenetic analysis demonstrates that this strain groups with other recent ZIKV isolates from the ongoing Latin American outbreaks (Fig. 1A). A comparison of the amino acid sequence of Paraiba_01/2015 with those of ZIKV strains obtained from human microcephaly cases reveals that it differs by 4, 3, and 7 amino acid positions from strains ZIKV2015, Natal RGN, and BeH823339, respectively (see Table S1 in the supplemental material). Interestingly, all of these differences were unique for individual viruses associated with microcephaly, and none of these changes were shared by two or more pathogenic strains.

### Development of the infectious cDNA clone of Paraiba_01/2015 strain of ZIKV.

The ICD of the Paraiba_01/2015 strain was generated by inserting full-length viral cDNA into the low-copy-number vector pACNR1811 (15) under the control of the eukaryotic RNA polymerase II (Pol II) cytomegalovirus (CMV) promoter (Fig. 1B), using a strategy described previously (16, 17). To simplify the assembly of the cDNA clone, a synonymous substitution, C→G, was introduced at nucleotide (nt) position 9887 of the viral genome, creating a unique site for BssHI restriction endonuclease. To ensure the release of the authentic 3′ end of the viral RNA, the hepatitis delta virus (HDV) ribozyme and RNA Pol II terminator sequence were inserted after the 3′ end of the ZIKV genome. To restrict plasmid toxicity during propagation of cDNA in *Escherichia coli* (strain MC1061), an intron sequence was inserted after nt position 2711 (nonstructural protein 1 [NS1] gene), generating cDNA plasmid ZIKV-*1*. Infectious virus corresponding to a consensus sequence of the Paraiba_01/2015 strain was recovered by transfection of the ZIKV-*1* cDNA plasmid into Vero cells. The rescued virus reached a titer of 3.1 × 10^6^ PFU/ml by day 3 after infection and produced plaques with diameters of 0.45 ± 0.04 mm in Vero cells on day 4 (Fig. 1C and D). The stability of ZIKV-*1* plasmid DNA (pDNA) in *E. coli* was further improved by inserting a second copy of the intron sequence after nt position 8882 (NS5 gene). The yield of the resulting ZIKV-*ICD* plasmid in *E. coli* was increased by approximately 50% (up to ~40 to 60 µg for 200 ml of an LB medium). The insertion of the second intron did not affect the kinetics of virus recovery in Vero cells (*P* = 0.465, 2-way analysis of variance [ANOVA]) or the plaque morphology of ZIKV-*ICD* compared to that of ZIKV-*1* virus (Fig. 1D).

**FIG 2:**
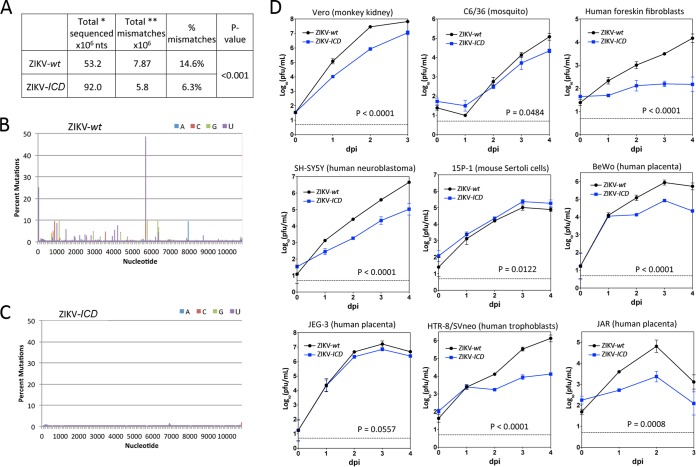
Quasispecies diversity and growth kinetics of ZIKV-*wt* and ZIKV-*ICD* viruses in different cell lines. (A) Summary of genome-wide deep-sequencing analysis of ZIKV-*wt* and ZIKV-*ICD* RNA. *, total number of nucleotides identified in quality-filtered reads; **, total number of mismatches in quality-filtered reads compared to the consensus sequence of the Paraiba_01/2015 strain. The frequencies of mismatches in ZIKV-*wt* and ZIKV-*ICD* were compared using the chi-square test. (B and C) Mutational profiles of ZIKV-*wt* (B) and ZIKV-*ICD* (C) RNAs. (D) Growth kinetics of ZIKV-*wt* and ZIKV-*ICD* in Vero, C6/36, human foreskin fibroblast, human neuroblastoma SH-SY5Y, mouse testis-derived Sertoli 15P-1, human trophoblast HTR-8/Neo, and human placenta-derived BeWo, JEG-3, and JAR cells. Each cell line was infected at an MOI of 0.01 PFU/cell in duplicate. Titers were determined by plaque assay in Vero cells and are presented as mean values ± standard deviations. Differences in growth kinetics were compared using 2-way ANOVA.

Due to a high mutation rate of viral RNA polymerases (18), it is postulated that RNA viruses exist not as a homogeneous entity but, rather, as a population (quasispecies) of mutant genomes which are centered around (but not always identical to) a consensus sequence (reviewed in references 19 and 20). Due to its long natural history, the parental Paraiba_01/2015 strain (designated ZIKV-*wt*) is expected to have a higher mutational diversity than a clone-derived ZIKV-*ICD* virus. This was confirmed by comparing the sequence heterogeneity of ZIKV-*wt* and ZIKV-*ICD* RNA genomes using deep-sequencing analysis. The overall frequency of mutations for ZIKV-*wt* was 2.3-fold higher than that of ZIKV-*ICD* (*P* < 0.001, two-tailed chi-square test) (Fig. 2A). Moreover, 61 nt positions of the ZIKV-*wt* genome contained polymorphisms at a frequency above 1%, and some of these positions contained polymorphisms in up to ~40% of all molecules (Fig. 2B; see also Table S2 in the supplemental material). In contrast, only a single position (nt 6861) of the ZIKV-*ICD* genome contained heterogeneity, and this variance occurred at a frequency of 1.37% (Fig. 2C; see also Table S3). Therefore, ZIKV-*wt* possesses a greater mutational diversity than the clone-derived ZIKV-*ICD* strain.

Quasispecies theory predicts that genetic diversity ensures higher robustness of a viral population (reviewed in references 20 and 21), which is understood here as the ability of virus to be adapted simultaneously to a wide range of conditions. Since ZIKV-*wt* exhibits greater genetic diversity than the clone-derived ZIKV-*ICD*, it is expected to be better adapted (as shown by faster replication) to diverse cell types. To test this hypothesis, we compared the growth kinetics of ZIKV-*ICD* and ZIKV-*wt* in a variety of cell lines infected at a multiplicity of infection (MOI) of 0.01 PFU/cell. The replication of ZIKV-*ICD* was substantially impaired compared to that of ZIKV-*wt* in the majority of cell lines, including cells of neuronal and placental origin that are targeted by ZIKV *in vivo* (Fig. 2D), supporting the role of genetic diversity in maintaining the replicative fitness of viral populations under changing conditions.

### Development of a recombinant ZIKV adapted for efficient replication in Vero cells.

**FIG 3:**
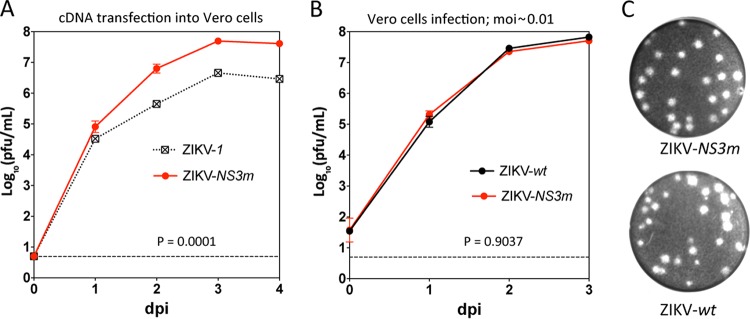
Effect of the NS3 Ser_356_Phe substitution on ZIKV fitness in Vero cells. (A) Growth kinetics of ZIKV-*1* and ZIKV-*NS3m* after plasmid DNA transfection into Vero cells. Samples for each time point from one biological replicate were titrated in Vero cells in duplicate, and results are presented as mean values ± standard deviations. (B) Multistep growth curves of ZIKV-*wt* and ZIKV-*NS3m* in Vero cells infected at an MOI of 0.01 PFU/cell. Titers are presented as mean values from 2 biological replicates ± standard deviations. Differences in growth kinetics were compared using 2-way ANOVA. (C) Plaque morphology of ZIKV-*NS3m* and ZIKV-*wt* in Vero cells at 4 dpi.

One of the primary uses of infectious cDNA clones is the development of live attenuated viruses applicable for vaccine research. However, the replication of ZIKV-*ICD* was found to be impaired in Vero cells, the cell line often used for vaccine production (Fig. 2D). This suggests that recombinant viruses developed on the basis of the ZIKV-*ICD* genome might be unstable during propagation in Vero cells, limiting the use of this clone for vaccine development. To overcome this restriction, ZIKV-*wt* was passaged 10 times in Vero cells, followed by sequencing of the plaque-purified viruses. Two of three selected clones contained a common nt substitution, C→T, at nt position 5680, leading to a Ser_356_Phe mutation in the NS3 protein. To demonstrate that this mutation increases the fitness of clone-derived virus in Vero cells, we introduced it into the ZIKV-*1* cDNA plasmid and generated the ZIKV-*NS3m* plasmid. The replication of ZIKV-*NS3m* virus recovered after cDNA plasmid transfection was significantly increased compared to that of ZIKV-*1* (Fig. 3A). More importantly, the replication of ZIKV-*NS3m* in Vero cells infected at an MOI of 0.01 PFU/cell was undistinguishable from that of ZIKV-*wt* (Fig. 3B). Interestingly, ZIKV-*NS3m* produced only large plaques in Vero cells (0.95 ± 0.12 mm). In contrast, ZIKV-*wt* generated plaques of various sizes (0.71 ± 0.23 mm) (Fig. 3C), consistent with deep-sequencing data that demonstrated high heterogeneity of ZIKV-*wt* (see Tables S2 and S4 in the supplemental material). In addition, despite the incorporation of mutations adapting virus to Vero cells, the growth of ZIKV-*NS3m* remained substantially impaired compared to that of ZIKV-*wt* in most cell lines, including human neuroblastoma and four different placenta-derived cell lines (see Fig. S1). This is another confirmation that the genetic diversity of ZIKV-*wt* may contribute to its higher robustness compared to that of clone-derived viruses.

## DISCUSSION

Contrary to single-stranded RNA viruses from many other families, the development of a reverse-genetics system for viruses belonging to the *Flavivirus* family has often been problematic due to toxicity of viral cDNA in bacterial systems. A number of approaches to resolve this issue have been explored, including (i) PCR-based approaches to generate infectious cDNA without cloning (22, 23), (ii) inactivation of cryptic promoters for bacterial RNA polymerase in the flavivirus genome using silent mutagenesis (24), (iii) splitting of viral cDNA for propagation in two or more plasmids before assembly of a full-length genome by *in vitro* ligation (25), (iv) insertion of linker sequences that disrupt the viral open reading frame and are removed prior to transcription (26), and (v) the use of homologous recombination in yeast (27). The most ubiquitous approach involves cloning a full-length viral cDNA into low-copy-number vectors, including plasmids (28), bacterial artificial chromosomes (29), and cosmids (30). In our experience, many of these approaches are laborious and time consuming and/or are not fully reliable.

In the present study, a stable cDNA clone of the epidemic Paraiba_01/2015 strain of ZIKV was constructed by placing intron sequences into the open reading frame of ZIKV (Fig. 1). Intron sequences disrupt the viral polyprotein open reading frame, causing premature translation termination. The insertion of a single copy of the intron sequence into the NS1 gene was sufficient for stable propagation of an ICD clone of the epidemic Paraiba_01/2015 strain in *E. coli* (Fig. 1C). Earlier, we observed that stabilization of cDNA clones for other flaviviruses (Rio Bravo, Langat, and Dengue type 4 viruses) in *E. coli* requires an additional copy of the intron sequence to be placed in the NS5 gene (N. Vasilakis, UTMB, Galveston, TX, and K. Tsetsarkin, NIAID, Bethesda, MD, unpublished data). The insertion of a second copy of the intron sequence into the NS5 gene of ZIKV does not interfere with rescue of infectious virus from the ICD clone of the Paraiba_01/2015 strain of ZIKV (ZIKV-*ICD*) but does increase the plasmid yield in *E. coli*. Therefore, to minimize the effects of possible strain-specific variations on ICD clone stability, we recommend adapting the 2-intron configuration for the development of a reverse-genetics system for other ZIKV isolates.

Launching virus replication through transfection with plasmid cDNA using the CMV promoter ensures the excision of the introns from the viral genome and significantly simplifies virus recovery compared to the procedures required by conventional T7 or SP6 RNA polymerase-based approaches. Some cell lines frequently used for the manufacture of vaccines (e.g., Vero cells) are notoriously difficult to transfect with RNA. Therefore, the ability to directly launch virus replication by plasmid cDNA transfection increases the range of cell lines suitable for ZIKV recovery and eliminates the need to rely on specific cell lines suitable for efficient RNA transfection. Nevertheless, the presence of intron sequences in the ZIKV ORF would make it difficult to modify our system into an RNA-based launching approach, which might be more convenient than DNA launching systems in some applications of viral reverse genetics, where rapid degradation of transfected material is desired.

Phylogenetic analysis showed that the Paraiba_01/2015 isolate is located in ZIKV clade Z, which contains six recently isolated ZIKV strains, including two human isolates (Natal RGN and BeH823339) from cases of congenital microcephaly (Fig. 1) (31, 32). Clade Z is defined by a single unique substitution, Met_349_Val, at the C-terminal end of the NS1 protein (amino acid position 1143 in the polyprotein) (see Table S1 in the supplemental material). Even though this substitution is absent in the third known microcephaly-associated ZIKV strain (ZKV2015), as well as in all other sequenced ZIKV strains, the high prevalence of microcephaly putatively caused by clade Z strains (33%) warrants further investigation of the potential role of the NS1 Met_349_Val substitution in fetus pathologies using reverse genetics. The ICD clone of Paraiba_01/2015 strain described here may be instrumental in this ongoing investigation.

The growth of virus derived from the ICD clone of Paraiba_01/2015 strain (ZIKV-*ICD*) was significantly impaired compared to the growth of ZIKV-*wt* in all cell lines except 15P-1 (mouse testis Sertoli cells). This is in agreement with the lower level of fitness of clone-derived virus of ZIKV strain FSS13025 in Vero and C6/36 cells compared to the fitness of the parental wild-type (wt) virus. Together with an earlier observation (14), our deep-sequencing data (Fig. 2B and C; see also Tables S2 and S3 in the supplemental material) support the hypothesis that high genetic diversity of the viral population contributes to higher replicative robustness of wild-type strains of ZIKV. Alternatively, a lower level of fitness for ZIKV-*ICD* might occur if the actual consensus sequence of Paraiba_01/2015, used for construction of ZIKV-*ICD*, does not accurately represent the population of ZIKV-*wt* sequences, where multiple epistatic mutations might independently increase the fitness of each individual genome in a population. However, the increased growth kinetics of ZIKV-*ICD* compared to the growth of ZIKV-*wt* in mouse testis-derived 15P-1 cells does not support this assumption (Fig. 2D). Since mice are not a natural host for ZIKV, it is unlikely that populations of ZIKV-*wt* would contain genomes with mutations selected for higher replication of ZIKV in these cells. These data indirectly demonstrate that additional epistatic mutations are not likely needed to achieve a level of ZIKV-*ICD* replication comparable to or greater than that of ZIKV-*wt.*

The deep-sequencing results indicate that mutations are not uniformly distributed across the ZIKV genome, and some nt positions contain substitutions at very high frequencies (Fig. 2B; see also Table S2 in the supplemental material). It is likely that the observed mutational profile reflects the natural passage history of ZIKV-*wt*, where individual substitutions were selected for better ZIKV fitness in a specific cell type (microenvironment) that is critical for viral transmission. However, this genetic heterogeneity might be somewhat restricted for vector-borne viruses whose life cycle involves replication in different hosts. The genetic diversity and a particular mutational profile of ZIKV may be repeatedly regenerated after the virus encounters tissue- and host-specific bottlenecks, as was shown for West Nile virus in different mosquito vectors (33, 34). Additionally, an established genetic diversity might be preserved in the viral population via coinfection of individual cells within the organism (35, 36). Future research is needed to establish approaches to accurately convey a specific heterogeneity profile of natural isolates to the clone-derived viruses. We are skeptical that current methods of random genome mutagenesis (see references 23, 36, and 37) will be appropriate for this task. Until this limitation is overcome, caution should be exercised during the interpretation of results provided by reverse genetic experiments in attempts to accurately predict the biology of natural viruses. Interestingly, some of the positions that had high variability in ZIKV-*wt* were also found to be variable among different ZIKV strains; however, the significance of that observation remains to be determined (see Table S2).

Since Vero cells are used for commercial production of biological materials (38), we developed a Vero cell-adapted ZIKV clone, ZIKV-*NS3m*, that may be used as a platform for the development of ZIKV vaccine candidates and therapeutics. The increased replication of ZIKV-*NS3m* in Vero cells compared to the replication of ZIKV-*ICD* was due to the incorporation of a single Ser_356_Phe substitution in the NS3 protein (Fig. 3). Interestingly, this substitution was present in the original ZIKV-*wt* at a frequency of 39.95%, which was the highest among all polymorphisms detected in ZIKV-*wt* (see Table S2 in the supplemental material). Since the Ser_356_Phe substitution increases the replication of ZIKV in Vero cells but not in any other cell lines, it is likely that this mutation was accumulated in ZIKV-*wt* during the two passages in Vero cells used for stock preparation. This observation is in agreement with the suggestion that the heterogeneity of ZIKV-*wt* reflects the natural passage history of Paraiba_01/2015.

In summary, we report an efficient reverse-genetics system for ZIKV isolated from the 2015 epidemic in Brazil, which will be used for studies of virus-host interaction and vaccine development. However, the complex role for genetic diversity in maintaining the replicative fitness of natural isolates of ZIKV, described here, suggests that caution should be exercised while interpreting the results of reverse-genetics experiments in attempts to accurately predict the biology of natural viruses.

## MATERIALS AND METHODS

### Cells.

Vero (African green monkey kidney) cells were grown in Opti-Pro medium (Invitrogen) supplemented with 50 µg/ml of gentamicin as previously described (39). For ZIKV infection, Vero cell medium was changed to complete Dulbecco's modified Eagle medium (DMEM [Invitrogen] supplemented with 10% fetal bovine serum [FBS] and 1× penicillin-streptomycin-glutamine solution [Invitrogen]). Mosquito-derived C6/36 cells (*Aedes albopictus*) and 15P-1 cells (mouse testis Sertoli cells; ATCC) were maintained in complete DMEM at 32°C and 5% CO_2_. Primary human foreskin fibroblasts (ATCC) and SH-SY5Y cells (human neuroblastoma, CRL-2266; ATCC) were maintained in complete DMEM at 37°C and 5% CO_2_. HTR-8/SVneo human trophoblasts (CRL3271; ATCC) and JAR human placenta cells (HTB-144; ATCC) were maintained in RPMI 1640 medium (ATCC) supplemented with 5% FBS and 1× penicillin-streptomycin solution (Invitrogen) at 37°C and 5% CO_2_. BeWo human placenta cells (CCL-98; ATCC) were maintained in Ham’s F-12K medium (ATCC) supplemented with 10% FBS and 1× penicillin-streptomycin-glutamine solution (Invitrogen) at 37°C and 5% CO_2_. JEG-3 human placenta cells (HTB-36; ATCC) were maintained in Eagle minimum essential medium (EMEM; ATCC) supplemented with 10% FBS and 1× penicillin-streptomycin solution at 37°C and 5% CO_2_.

### Virus.

The Paraiba_01/2015 strain of ZIKV was originally isolated in the state of Paraiba (Brazil) in 2015 from a serum sample of a febrile female and was kindly provided by Pedro Vasconcelos, Instituto Evandro Chagas, Brazil. The virus initially had one passage in Vero cells in our laboratory, and a working stock of the virus (ZIKV-*wt*) was generated following a second passage in Vero cells grown in OptiPro medium supplemented with 2% FBS and 2 mM l-glutamine. Viral RNA was extracted from the working stock using a QIAamp viral RNA minikit (Qiagen, Valencia, CA), followed by cDNA production using SuperScript III reverse transcriptase (Invitrogen Life Technologies) and random hexamer primers. Overlapping cDNA fragments were amplified using *Taq* DNA polymerase and sequenced using a 3730 DNA analyzer (Applied Biosystems, Foster City, CA). To determine the 5′ and 3′ termini of the ZIKV genome, viral RNA was treated with tobacco acid pyrophosphatase (Ambion) and ligated using T4 RNA ligase (Ambion). The region flanking the junction site of the ligated RNA was amplified using a Transcriptor one-step reverse transcriptase PCR (RT-PCR) kit (Roche) and sequenced. The full-length ZIKV genome sequence was assembled and edited with Sequencher version 5.3 (Gene Codes, Ann Arbor, MI).

### Infectious clone construction.

To generate the infectious cDNA (ICD) clone, overlapping cDNA fragments of the Paraiba_01/2015 genome were amplified using high-fidelity Phusion DNA polymerase (NEB) and cloned individually or in combination into the low-copy-number vector pACNR1811 (15). All plasmids were assembled using conventional cloning methods (40). To simplify the assembly of the cDNA, a synonymous substitution, C→G, was introduced at nt position 9887 of the viral genome, creating a unique site for BssHI restriction endonuclease. Eukaryotic RNA polymerase II (Pol II) cytomegalovirus (CMV) promoter was PCR amplified from the pCMV-SPORT6 plasmid (Invitrogen, Carlsbad, CA) and was introduced upstream from the 5′ end of ZIKV genome (Fig. 1B). An antigenomic HDV ribozyme and an RNA Pol II terminator sequence were amplified from plasmid D4 (17) and inserted downstream from the 3′ end of the ZIKV genome. The intron sequence was amplified by PCR from plasmid D4 and inserted after nt position 2711 (NS1 gene) to generate plasmid ZIKV-*1*. A second copy of the intron sequence was inserted into ZIKV-*1* cDNA after nt position 8882 (NS5 gene) of the ZIKV genome to generate plasmid ZIKV-*ICD*. A Vero adaption mutation (C→T) was introduced into ZIKV-*1* cDNA at nt position 5680 of the ZIKV genome (NS3 gene) to generate ZIKV-*NS3m* carrying a Ser_356_Phe substitution in the NS3 protein. All intermediate and final DNA plasmids were propagated in the MC1061 strain of *E. coli*.

### DNA transfection, virus recovery, and titration.

Viruses were recovered from plasmid DNA using a method described previously (17). Briefly, Vero cells were seeded into a 12.5-cm^2^ flask in complete DMEM (DMEM supplemented with 10% FBS and 1× penicillin-streptomycin-glutamine solution) and incubated for 24 h at 37°C and 5% CO_2_. On the following day, the cell culture medium was replaced with Opti-MEM (Invitrogen), and the cells were transfected with 2.5 µg of pDNA using Lipofectamine 2000 reagent (Invitrogen). Cells were washed twice with Opti-MEM and maintained at 37°C and 5% CO_2_ for 4 days in 5 ml of complete DMEM. Cell culture aliquots (0.5 ml) were collected daily and stored at −80°C. The virus titer was determined by plaque assay in Vero cells using a 10-fold serial dilution of samples in duplicate in complete Opti-Pro medium (Opti-Pro supplemented with 2% FBS, 2 mM l-glutamine, 50 µg/ml gentamicin). Vero cells in 24-well plates were incubated with 0.1 ml of diluted viruses for 1 h at 37°C, and 1 ml of Opti-MEM containing 1% methylcellulose (Invitrogen), 2% FBS, 2 mM l-glutamine, and 50 µg/ml gentamicin was added to each well. Plates were incubated at 37°C and 5% CO_2_ for 4 days, fixed for 20 min with 100% methanol, and stained with 0.5% crystal violet solution.

### Replication kinetics of parental and clone-derived ZIKV in cells.

The growth kinetics of parental and cDNA-derived viruses in various cell lines were determined as described previously (17). For each cell line, 1 × 10^6^ cells were seeded into 12.5-cm^2^ flasks in appropriate maintenance medium for 24 h prior to virus infection. On the following day, viruses were diluted in complete DMEM to a concentration of 4 × 10^4^ PFU/ml (virus titers were determined on Vero cells), and 0.5 ml of diluted virus was used to infect cells in duplicate flasks for 1 h at 32°C (C6/36 and 15P-1 cells) or at 37°C (for all other cell lines). The cells were washed two times with fresh maintenance medium and then supplemented with 3 ml of the appropriate maintenance medium and incubated at 32°C (C6/36 and 15P-1 cells) or at 37°C (for all other cell lines) and in 5% CO_2_ for 3 or 4 days. Aliquots of cell culture medium (0.3 ml) were harvested daily and stored at −80°C until virus titration. Differences in virus replication kinetics were compared using 2-way ANOVA analysis implemented in Prism 6 software (La Jolla, CA).

### Deep-sequencing analysis.

Vero cell supernatants were harvested on day 4 posttransfection with ZIKV-*ICD* or ZIKV-*NS3m* plasmid DNA and clarified by centrifugation at 2,100 × *g* for 5 min. It was shown that packaging of flavivirus genomes into virus particles occurs only during active genomic RNA replication (41), which precludes the accumulation of nonreplicating ZIKV genomes in the supernatant. To reduce cellular RNA background and plasmid contamination, the ZIKV-*wt* (stock solution) and ZIKV-*ICD* or ZIKV-*NS3m* viruses were treated with micrococcal nuclease (NEB) for 2 h at 37°C. The reaction was terminated by adding EGTA (pH 8.0) to a final concentration of 36 mM. Total RNA was isolated from 140 µl of micrococcal nuclease-treated samples using a QIAamp viral RNA minikit (Qiagen, Valencia, CA) according to the manufacturer’s instructions. To prepare the DNA library for Illumina sequencing, 0.25 µg of total RNA was fragmented using a Focused-ultrasonicator (Covaris) to generate fragments of around 500 nt. To prepare the DNA library from the RNA fragments, the NEBNext mRNA library prep master mix set for Illumina (New England Biolabs) was used according to the manufacturer’s protocol. Briefly the fragmented RNA was reverse transcribed and the second-strand cDNA was synthesized, resulting in the DNA fragments being ligated to Illumina paired-end adaptors. These were then amplified using 12 cycles of PCR with multiplex indexed primers, and the products purified on magnetic beads (Agencourt AMPure PCR purification system; Beckman Coulter). After analyzing the libraries for size and quality (BioAnalyzer; Agilent Technologies, Inc.), deep sequencing was performed using MiSeq (Illumina), producing 250-nucleotide paired-end reads. The raw sequencing reads were analyzed using SWARM custom software.

### Phylogenetic studies.

The complete coding sequences of the Paraiba_01/2015 strain and 60 representative ZIKV strains downloaded from GenBank were aligned using MUSCLE (42), followed by manual adjustment in Se-Al (available at http://tree.bio.ed.ac.uk/software/seal/) to preserve codon homology (a total of 61 genome sequences were used in the analysis). This led to an alignment length of 10,270 nt. The phylogenetic tree was constructed using the maximum-likelihood method implemented in PAUP*, version 4.0b (43), utilizing the best-fit model estimate of ModelTest (44). The bootstrap values were estimated using bootstrap resampling (1,000 replications) of the neighbor-joining algorithm, and input genetic distances were determined using the maximum-likelihood substitution model.

### Sequence accession number.

The full-length genome sequence of Paraiba_01/2015 was deposited in GenBank under accession number KX280026. The complete sequence of the ZIKV-*ICD* plasmid was deposited in GenBank with appropriate annotations under accession number KX576684.

## SUPPLEMENTAL MATERIAL

Figure S1  Growth kinetics of ZIKV-*wt* and ZIKV-*NS3m* viruses in different cell lines. Growth kinetics of ZIKV-*wt* and ZIKV-NS3m in Huh7, C6/36, human foreskin fibroblast, human neuroblastoma SH-SY5Y, mouse testis-derived Sertoli 15P-1, human trophoblast HTR-8/Neo, and human placenta-derived BeWo, JEG-3, and JAR cells. Each cell line was infected at an MOI of 0.01 PFU/cell in duplicate. Titers were determined by plaque assay in Vero cells and are presented as mean values ± standard deviations. Differences in growth kinetics were compared using 2-way ANOVA (Huh7 cells were maintained in complete DMEM medium). Download Figure S1, PDF file, 0.4 MB

Table S1  Amino acid differences between Paraiba_01/2015 and ZIKV strains associated with human congenital microcephaly. Table S1, PDF file, 0.1 MB

Table S2  Mutational profile of ZIKV-*wt* genome. Positions within the ZIKV-*wt* genome that contained single nucleotide polymorphisms (SNPs) at frequencies above 1% of quality-filtered reads are indicated, along with the corresponding amino acid substitutions. “Informative” denotes sites that provide information for phylogenetic construction; “Uninformative” denotes sites that in the phylogenetic alignment have no or only one ZIKV strain with a different residue; “State” shows how many states (different nucleotides) occur in that position in the alignment; and “Steps” shows how many steps of mutations in total occur in the tree for that position. Table S2, PDF file, 0.3 MB

Table S3  Mutational profile of ZIKV-*ICD* genome. Positions within the ZIKV-*ICD* genome that contained single nucleotide polymorphisms (SNPs) at frequencies above 1% of quality-filtered reads are indicated, along with the corresponding amino acid substitutions. Table S3, PDF file, 0.1 MB

Table S4  Mutational profile of ZIKV-*NS3m* genome. Positions within the ZIKV-*NS3m* genome that contained single nucleotide polymorphisms (SNPs) at frequencies above 1% of quality-filtered reads are indicated, along with the corresponding amino acid substitutions. Table S4, PDF file, 0.1 MB
