# Supplementary material for: A Full-Length Infectious cDNA Clone of Zika Virus from the 2015 Epidemic in Brazil as a Genetic Platform for Studies of Virus-Host Interactions and Vaccine Development
Source: mBio. 2016 Aug 23;7(4):e01114-16. doi: 10.1128/mBio.01114-16 (PMC4999549; doi:10.1128/mBio.01114-16)
Supplement: Figure S1 — Growth kinetics of ZIKV-wt and ZIKV-NS3m viruses in different cell lines. Growth kinetics of ZIKV-wt and ZIKV-NS3m in Huh7, C6/36, human foreskin fibroblast, human neuroblastoma SH-SY5Y, mouse testis-derived Sertoli 15P-1, human trophoblast HTR-8/Neo, and human placenta-derived BeWo, JEG-3, and JAR cells. Each cell line was infected at an MOI of 0.01 PFU/cell in duplicate. Titers were determined by plaque assay in Vero cells and are presented as mean values ± standard deviations. Differences in growth kinetics were compared using 2-way ANOVA (Huh7 cells were maintained in complete DMEM medium). Download [file mbo004162955sf1.pdf]

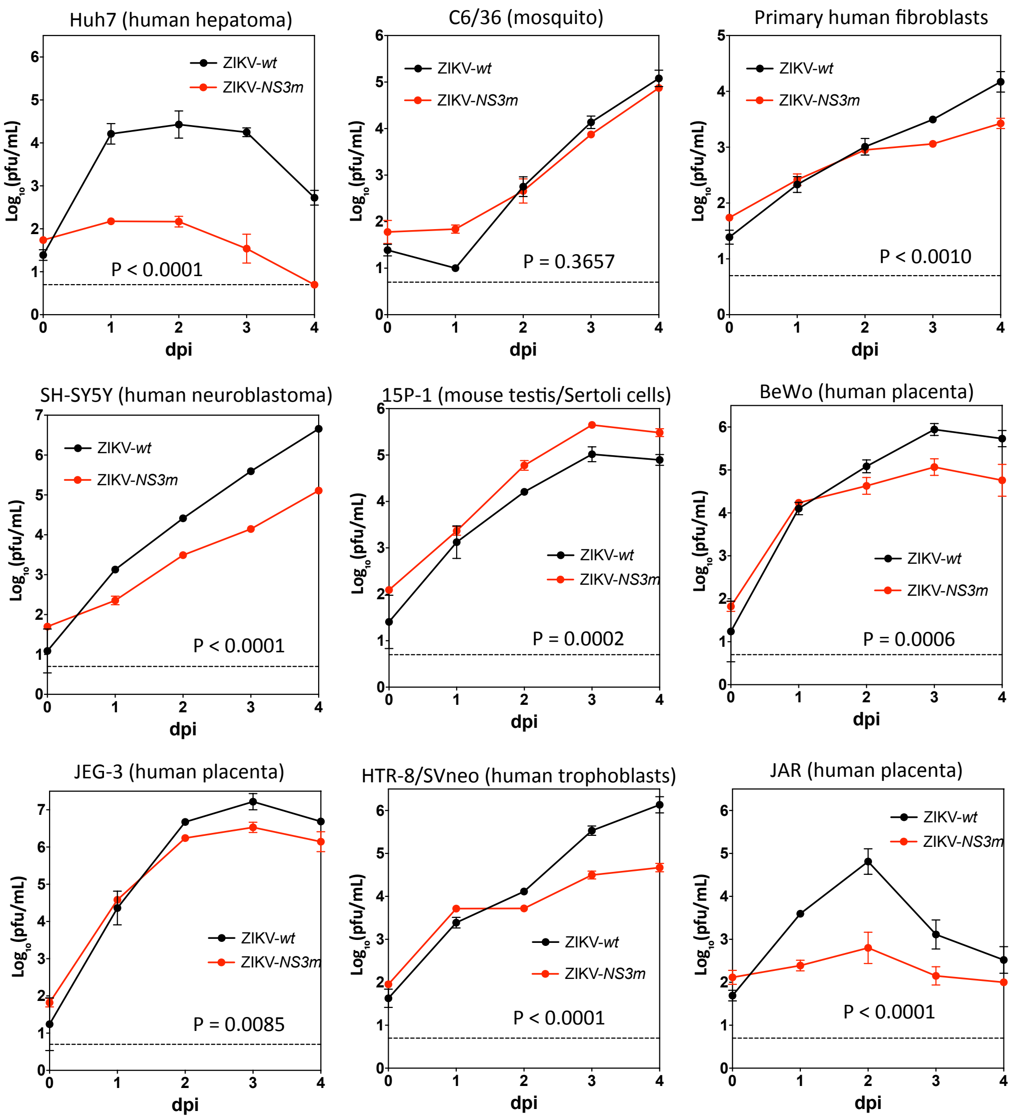

Figure S1

Supplementary Figure S1. Growth kinetics of ZIKV-*wt* and ZIKV-*NS3m* viruses in different cell lines.

Growth kinetics of ZIKV-*wt* and ZIKV-*NS3m* in Huh7, C6/36, human foreskin fibroblast, human neuroblastoma SH-SY5Y, mouse testis-derived Sertoli 15P-1, human trophoblast HTR-8/Neo, and human placenta-derived BeWo, JEG-3, and JAR cells. Each cell line was infected at an MOI of 0.01 pfu/cell in duplicate. Titers were determined by a plaque assay in Vero cells and presented as mean values ± standard deviation. Differences in growth kinetics were compared using 2-way ANOVA.

(Huh7 cells were maintained in the complete DMEM medium).
