# Supplementary material for: A Full-Length Infectious cDNA Clone of Zika Virus from the 2015 Epidemic in Brazil as a Genetic Platform for Studies of Virus-Host Interactions and Vaccine Development
Source: mBio. 2016 Aug 23;7(4):e01114-16. doi: 10.1128/mBio.01114-16 (PMC4999549; doi:10.1128/mBio.01114-16)
Supplement: Table S1 — Amino acid differences between Paraiba_01/2015 and ZIKV strains associated with human congenital microcephaly. [file mbo004162955st1.pdf]

Supplementary Table S1. Amino acid differences between a Paraiba_01/2015 strain and ZIKV strains associated with human congenital microcephaly.

Amino acid position in polyprotein	Protein	Zika virus strains and GenBank accession number			
		ZKV2015 KU497555	Natal RGN KU527068	BeH823339 KU729217	Paraiba_01/2015 KX280026
337	M	Thr	Thr	**Ser**	Thr
354	E	Ser	Ser	**Thr**	Ser
358		Met	Met	**Ile**	Met
545		Val	Val	**Ala**	Val
550		**Thr**	Ser	Ser	Ser
940	NS1	Lys	**Glu**	Lys	Lys
984		Gly	Gly	**Glu**	Gly
1027		Thr	**Ala**	Thr	Thr
1143		**Met**	Val	Val	Val
1259	NS2A	**Phe**	Leu	Leu	Leu
1404	NS2B	Met	Met	**Ile**	Met
2509	NS5	Thr	**Ile**	Thr	Thr
2800		Asn	Asn	**Asp**	Asn
2831		**Val**	Glu	Glu	Glu
