# Supplementary material for: A Full-Length Infectious cDNA Clone of Zika Virus from the 2015 Epidemic in Brazil as a Genetic Platform for Studies of Virus-Host Interactions and Vaccine Development
Source: mBio. 2016 Aug 23;7(4):e01114-16. doi: 10.1128/mBio.01114-16 (PMC4999549; doi:10.1128/mBio.01114-16)
Supplement: Table S2 — Mutational profile of ZIKV-wt genome. Positions within the ZIKV-wt genome that contained single nucleotide polymorphisms (SNPs) at frequencies above 1% of quality-filtered reads are indicated, along with the corresponding amino acid substitutions. “Informative” denotes sites that provide information for phylogenetic construction; “Uninformative” denotes sites that in the phylogenetic alignment have no or only one ZIKV strain with a different residue; “State” shows how many states (different nucleotides) occur in that position in the alignment; and “Steps” shows how many steps of mutations in total occur in the tree for that position. [file mbo004162955st2.pdf]

Supplementary Table S2. Mutational profile of ZIKV-*wt* genome.

Positions within ZIKV-*wt* genome that contained single nucleotide polymorphisms (SNP) at a frequency above 1% of quality-filtered reads and corresponding amino acid substitutions are indicated. 'Informative' sites that provide information for phylogenetic construction. 'Uninformative' - sites that in the phylogenetic alignment have none or only one ZIKV strain with a different residue. 'State' shows how many states (different nucleotide) in that position in the alignment. 'Steps' shows how many steps of mutations in total in the tree for that position.

	A	B	C	D	E	F	G	H	I	J	K
1	Nucleotide	Base	Mutation	Gene	Amino acid	Reference amino acid	Mutant amino acid	% Mutants		States	Steps
2	132	G	C	capsid protein C	9	Gly	Arg	1.01	Uninformative	1	
3	137	A	G	capsid protein C	10	Gly		1.04	Uninformative	1	
4	142	G	C	capsid protein C	12	Arg	Pro	1.01	Uninformative	1	
5	147	G	U	capsid protein C	14	Val	Phe	1.12	Uninformative	1	
6	148	U	G	capsid protein C	14	Val	Gly	1.02	Uninformative	1	
7	149	C	U	capsid protein C	14	Val		1.07	Uninformative	1	
8	152	U	A	capsid protein C	15	Asn	Lys	1.16	Uninformative	1	
9	153	A	U	capsid protein C	16	Met	Leu	1.17	Uninformative	1	
10	154	U	A	capsid protein C	16	Met	Lys	1.17	Uninformative	1	
11	162	C	A	capsid protein C	19	Arg	Ser	1.05	Uninformative	1	
12	170	A	U	capsid protein C	21	Val		1.04	Uninformative	1	
13	189	G	U	capsid protein C	28	Gly	Trp	1.13	Uninformative	1	
14	693	A	G	protein pr	74	Thr	Ala	4.23	Uninformative	1	
15	748	G	A	protein pr	92	Arg	Lys	1.77	Uninformative	1	
16	793	C	U	small envelope protein M	14	Thr	Met	1.90	Uninformative	1	
17	798	U	C	small envelope protein M	16	Ser	Pro	4.71	Uninformative	1	
18	861	U	C	small envelope protein M	37	Phe	Leu	9.40	Uninformative	2	1
19	970	C	U	small envelope protein M	73	Ala	Val	8.54	Uninformative	1	
20	1087	A	G	envelope protein E	37	Asp	Gly	9.58	Uninformative	1	
21	1126	U	C	envelope protein E	50	Val	Ala	1.42	Uninformative	1	
22	1449	C	U	envelope protein E	158	His	Tyr	5.77	Uninformative	2	1
23	1887	U	A	envelope protein E	304	Ser	Thr	2.81	Uninformative	1	
24	1921	C	U	envelope protein E	315	Thr	Ile	2.73	Uninformative	1	
25	1933	C	U	envelope protein E	319	Ala	Val	1.88	Uninformative	1	
26	2178	C	U	envelope protein E	401	His	Tyr	3.04	Uninformative	1	
27	2478	G	A	envelope protein E	501	Ala	Thr	3.06	Uninformative	1	
28	2537	U	C	nonstructural protein NS1	16	Gly		2.72	Informative	2	1
29	2781	U	C	nonstructural protein NS1	98	Trp	Arg	1.40	Uninformative	1	
30	2781	U	G	nonstructural protein NS1	98	Trp	Gly	1.23	Uninformative	1	
31	2783	G	U	nonstructural protein NS1	98	Trp	Cys	1.38	Uninformative	1	
32	2786	A	U	nonstructural protein NS1	99	Arg	Ser	3.04	Uninformative	1	
33	2914	A	G	nonstructural protein NS1	142	Glu	Gly	2.01	Uninformative	1	
34	2925	A	G	nonstructural protein NS1	146	Lys	Glu	4.44	Informative	2	3
35	3282	A	G	nonstructural protein NS1	265	Lys	Glu	1.84	Uninformative	2	
36	3535	U	C	nonstructural protein NS1	349	Val	Ala	4.20	Uninformative	1	
37	3895	C	U	nonstructural protein NS2A	117	Ala	Val	2.69	Uninformative	1	
38	3930	A	C	nonstructural protein NS2A	129	Ile	Leu	2.64	Informative	2	2
39	4015	C	U	nonstructural protein NS2A	157	Ala	Val	5.59	Uninformative	1	
40	4184	C	U	nonstructural protein NS2A	213	Asn		7.47	Informative	2	2
41	4338	A	G	nonstructural protein NS2B	39	Ile	Val	3.96	Uninformative	1	
42	4718	A	G	nonstructural protein NS3	35	Gln		1.91	Informative	2	1
43	5676	C	U	nonstructural protein NS3	355	His	Tyr	1.91	Informative	2	1
44	5680	C	A	nonstructural protein NS3	356	Ser	Tyr	8.56	Uninformative	1	
45	5680	C	U	nonstructural protein NS3	356	Ser	Phe	39.95	Uninformative	1	
46	5693	U	C	nonstructural protein NS3	360	Val		9.14	Informative	2	2
47	5744	A	G	nonstructural protein NS3	377	Thr		10.13	Uninformative	1	
48	6212	G	U	nonstructural protein NS3	533	Val		2.37	Uninformative	1	
49	6305	U	G	nonstructural protein NS3	564	Asp	Glu	9.80	Uninformative	1	
50	6373	A	G	nonstructural protein NS3	587	Lys	Arg	6.32	Uninformative	1	
51	6374	A	U	nonstructural protein NS3	587	Lys	Asn	4.17	Informative	2	1
52	6861	C	A	protein 2K	6	Gln	Lys	1.08	Uninformative	1	
53	6983	A	G	nonstructural protein NS4B	23	Gly		1.38	Informative	2	3
54	7847	A	C	RNA-dependent RNA polymera	60	Ala		1.54	Uninformative	1	
55	7943	C	A	RNA-dependent RNA polymera	92	Ala		9.20	Uninformative	2	
56	8708	C	U	RNA-dependent RNA polymera	347	Thr		3.01	Informative	3	2
57	9539	U	C	RNA-dependent RNA polymera	624	Asn		1.37	Informative	2	2
58	10310	G	A	RNA-dependent RNA polymera	881	Lys		1.02	Informative	2	1
59	10694	A	G	non-coding				1.79			
60	10700	G	A	non-coding				1.17			
61	10703	U	A	non-coding				1.01			
62	10735	A	G	non-coding				1.01			
