# Supplementary material for: A Full-Length Infectious cDNA Clone of Zika Virus from the 2015 Epidemic in Brazil as a Genetic Platform for Studies of Virus-Host Interactions and Vaccine Development
Source: mBio. 2016 Aug 23;7(4):e01114-16. doi: 10.1128/mBio.01114-16 (PMC4999549; doi:10.1128/mBio.01114-16)
Supplement: Table S3 — Mutational profile of ZIKV-ICD genome. Positions within the ZIKV-ICD genome that contained single nucleotide polymorphisms (SNPs) at frequencies above 1% of quality-filtered reads are indicated, along with the corresponding amino acid substitutions. [file mbo004162955st3.pdf]

Supplementary Table S3. Mutational profile of ZIKV-*ICD* genome.

A position within ZIKV-*ICD* genome that contained single nucleotide polymorphism (SNP) at frequency above 1% of quality-filtered reads and corresponding amino acid substitution are indicated.

	A	B	C	D	E	F	G	H	I
1	Nucleotide	Base	Mutation	Gene	Amino acid	Reference amino acid	Mutant amino acid	% Mutants	
2	6861	C	A	protein 2K	6	Gln	Lys	1.37	
3									
