# Supplementary material for: A Full-Length Infectious cDNA Clone of Zika Virus from the 2015 Epidemic in Brazil as a Genetic Platform for Studies of Virus-Host Interactions and Vaccine Development
Source: mBio. 2016 Aug 23;7(4):e01114-16. doi: 10.1128/mBio.01114-16 (PMC4999549; doi:10.1128/mBio.01114-16)
Supplement: Table S4 — Mutational profile of ZIKV-NS3m genome. Positions within the ZIKV-NS3m genome that contained single nucleotide polymorphisms (SNPs) at frequencies above 1% of quality-filtered reads are indicated, along with the corresponding amino acid substitutions. [file mbo004162955st4.pdf]

Supplementary Table S4. Mutational profile of ZIKV-*NS3m* genome.

A position within ZIKV-*NS3m* genome that contained single nucleotide polymorphism (SNP) at a frequency above 1% of quality-filtered reads, and corresponding amino acid substitution are indicated.

Nucleotide	Base	Mutation	Gene	Amino acid	Reference amino acid	Mutant amino acid	% Mutants
6861	C	A	protein 2K	6	Gln	Lys	1.27
